# Supplementary material for: Visual impairment in pseudoexfoliation from four tertiary centres in India
Source: PLoS One. 2020 May 29;15(5):e0233268. doi: 10.1371/journal.pone.0233268 (PMC7259498; doi:10.1371/journal.pone.0233268)
Supplement: S1 Table — (DOCX) [file pone.0233268.s001.docx]

Table S1: Baseline visual acuity and final visual acuity of patients with unilateral or bilateral pseudoexfoliation at different stages (see methods for detailed description of visual acuity groups 0-4).

| Baseline  vision*  % | 0 | 1 | 2 | 3 | 4 | Vision at 1 year*  % | 0 | 1 | 2 | 3 | 4 |
| --- | --- | --- | --- | --- | --- | --- | --- | --- | --- | --- | --- |
| PXF n=3024 | | | | | | | | | | | |
| Unilateral | 9.4 | 4.4 | 3 | 9.05 | 23.6 | Unilateral | 6.3 | 4.3 | 3.45 | 8.7 | 27.15 |
| Bilateral | 12.8 | 6.2 | 4.5 | 9.05 | 18 | Bilateral | 10.3 | 4.5 | 9.7 | 13.5 | 12.1 |
| PXF with OHT n=144 | | | | | | | | | | | |
| Unilateral | 7.4 | 1.3 | 0 | 11.1 | 21.9 | Unilateral | 7.2 | 2.35 | 0 | 9.8 | 30.6 |
| Bilateral | 14.1 | 3.6 | 7.7 | 9.4 | 23.5 | Bilateral | 8.8 | 2.75 | 7.1 | 20.3 | 11.1 |
| PXG n=1804 | | | | | | | | | | | |
| Unilateral | 9.8 | 3.2 | 2.3 | 10.15 | 24.5 | Unilateral | 8.2 | 3.4 | 2.3 | 8.3 | 14.7 |
| Bilateral | 15.8 | 6.2 | 3.75 | 8.6 | 15.7 | Bilateral* | 14.6 | 18.4 | 8.3 | 1.4 | 20.4 |

*-8eyes with bilateral PXF without reproducible visual acuity not included; PXF- Pseudoexfoliation; OHT-Ocular hypertension; :PXG-Pseudoexfoliation glaucoma
