# Supplementary material for: Visual impairment in pseudoexfoliation from four tertiary centres in India
Source: PLoS One. 2020 May 29;15(5):e0233268. doi: 10.1371/journal.pone.0233268 (PMC7259498; doi:10.1371/journal.pone.0233268)
Supplement: S2 Table — (DOCX) [file pone.0233268.s002.docx]

Table S2: Characteristics of eyes with pseudoexfoliation and secondary glaucoma across 4 tertiary centres

| N=96 | Descriptive summary |
| --- | --- |
| Baseline IOP (mm Hg) | 20±11.1 |
| Age (years) | 65±7.9 |
| Number of eyes on medications | PXF-2  Non-PXF-53  1-19,2-21,3-9,4-6 |
| Diagnosis | Lens induced-28 (22 phacomorphic, 6 phacolytic)  Angle closure 56  Other secondary glaucoma-12 |
| Proportion of OHT:PXG (all centres) | 2.8:36.2 |
| South India (%) | 2.1:29.5 |
| East India (%) | 2.9:29.04 |
| Absolute blindness rate for all centres at  Baseline visit  Final visit | 28.2%  21.9% |

PXF-Pseudoexfoliation; IOP-Intraocular pressure, *-see text methods for detailed description of visual acuity groups;
