# Supplementary material for: Visual impairment in pseudoexfoliation from four tertiary centres in India
Source: PLoS One. 2020 May 29;15(5):e0233268. doi: 10.1371/journal.pone.0233268 (PMC7259498; doi:10.1371/journal.pone.0233268)
Supplement: S3 Table — (DOCX) [file pone.0233268.s003.docx]

Table S3: Factors influencing higher risk of blindness in patients with pseudoexfoliation

| Independent variables | OR (95%CI) | p value |
| --- | --- | --- |
| Age | 0.97(0.95-0.98) | **0.001** |
| Laterality | 0.74(0.59-0.93) | **0.01** |
| Higher baseline IOP | 0.98(0.96-099) | **0.04** |
| VFI | 1.03(0.99-1.008) | 0.08 |
| Stage of PXF | 0.99 (0.54-1.12) | 0.8 |

IOP-Intraocular pressure; VFI-Visual field index, PXF-Pseudoexfoliation
