# Supplementary material for: Visual impairment in pseudoexfoliation from four tertiary centres in India
Source: PLoS One. 2020 May 29;15(5):e0233268. doi: 10.1371/journal.pone.0233268 (PMC7259498; doi:10.1371/journal.pone.0233268)
Supplement: S4 Table — (DOCX) [file pone.0233268.s004.docx]

Table S4: Surgeries done with complications and final outcome in patients with pseudoexfoliation in 4 tertiary centres

| Surgery done | Number of eyes | Complications |
| --- | --- | --- |
| IOL explantation | 11 | - |
| Phacoemulsification+trabeculectomy | 39 | Raised IOP and failure in 4 eyes |
| Trabeculectomy | 111 | - |
| Phacoemulsification | 458 | Zonular dialysis in 3 eyes |
| Small incision cataract surgery | 1375 | - |
| Small incision cataract surgery +anterior vitrectomy | 1 | - |
| Lacrimal surgery | 34 | Transient bleeding in 2 patients |
| Small incision cataract surgery +Trabeculectomy | 29 | Failure in 3 cases |
| Phacoemulsification+trabeculectomy+mitomyicn-C | 5 | - |
| Incision biopsy | 2 | - |
| Zaltrap(ziv-aflibercept)injections | 5 | - |
| Anterior vitrectomy | 33 | - |
| Pars plana vitrectomy (for subluxated or dislocated lens, vitreous hemorrhage, suspected infectious etiology) | 22 | Loss of vision-3  Hyphema transient-9  Disc pallor-2 |
| Extracapsular cataract extraction | 6 | - |
| Trans-scleral cyclophotocoagulation | 5 | - |
| Vitreous biopsy | 7 | - |
| Pterygium resection with autograft | 10 | - |
| Conjunctival resection withBCL | 5 | Perforation requiring PK in 3 eyes |
| Conjunctival resection with amniotic membrane | 1 | - |
| Cryotherapy and tumor resection (for ocular surface carcinoma) | 1 | - |
| IOAB (for established or suspected endophthalmitis) | 11 | - |

IOL-Intraocular lens, IOA-Intraocular antibiotics; BCL-bandage contact lens
